# Annotations

**Published:** 1891-07-18

**Authors:** 


### Annotations.

"Birch Rods by Post, a Shilling."

"Birchrods, by post, a shilling, well packed, with advice as to unruly children." Apply, &c. A weekly newspaper of good repute publishes this "swishing" advertisement; and the vendor of the shilling birch rods is a lady, and an unmarried one too. Who shall say after this that discipline is not advancing in seven-league boots? We dare not inform our youthful readers where this tremendous young lady lives who provides "improvers" at a shilling apiece and sends them by post. They would be wanting to take train by hundreds to get a peep at her round the nearest corner. Wicked boys might wish to do something even more desperate. Perhaps some of them would essay to pierce the darkness of the cellars of her dwelling, like nineteenth century Guy Fawkeses, and make a desperate attempt to blow her up with gunpowder. Some time ago a young medical graduate was consulted, in the absence of his principal, about a little girl of three or four, who was said to suffer periodically from fits of uncontrollable excitement. The old doctor had been in the habit of recommending soothing words and bromide of potassium. "Give her a whipping," said the terrible young man. The tender-hearted parents were disgusted with such brutality, and declined to see the young man any more. They continued the "soothing words with bromide" for two or three years longer, until the little creature became positively unbearable. Then the despised "whipping" was tried, and with the happiest results. Two or three applications of the treatment wrought a complete and permanent cure. Is Miss ——, who sells birch rods at a shilling, one of the maiden aunts of that "cured" little girl? Perhaps after all the advertisement is only a grim joke. Possibly there is no Miss —— at C——. We cannot help hoping, however, that it is really *bonâ fide*. Birch rods are exceedingly seasonable remedies occasionally, and much more speedily and thoroughly efficacious than bromide of potassium, in properly selected cases. If Miss —— will only provide thoroughly genuine articles, strong and flexible, she will deserve as much success and fortune as many another patent medicine vendor. Notwithstanding our severe medical orthodoxy, we wish her all success in her benevolent and widely useful enterprise.

Do Jews Ever Plough?

This question may be considered as equivalent to a larger one, namely, "Do Jews ever take up hard manual work, except when they are compelled?" The enquiry is important. We are coming to a time, if we have not already reached it, when those peculiar endowments which make for survival will be the most significant and successful of all human endowments. In other words, advantages of country, of political government, of family, and even of wealth, will tell less and less, and advantages of adaptability, courage, self-denial, and unyielding persistence will tell more and more. Strokes of luck are beginning to be things of the past. A *coup d'etat* is becoming less and less easy in small things as well as in great. Men have nowadays to win reputation by slow perseverance before they can wear it, and they have to earn fortune by capacity and diligence before they can enjoy it. Such being the undoubted conditions of modern life, it is important to consider what races possess the necessary qualifications for success in the largest measure; and how it may be possible to discover the deficiencies of our own fellow countrymen, and to develop in them the qualities that are feeble. The Jews, it is said, are as a race financiers and traders by preference, and are successful in finance because of their peculiar natural aptitude. The question is, are the Jews successful as financiers and traders because of special gifts in that direction, or because of their general brain capacity, their diligence, and their perseverance? The fact seems to be that the Jew is no more a born financier than he is a born farmer. He succeeds as well at farming as at finance, and as well at finance as at farming; and he succeeds at each for the same reason, namely, that he gives to each all the earnestness and capacity which he possesses, and he sticks to each with unyielding perseverance until he compels success. A strange circumstance is reported by Baron de Hirsch, whose name is just now in everybody's mouth on account of his great benevolence to his own people. Baron de Hirsch tells us that whilst English and French Christians are leaving the Argentine Republic in despair because they cannot make agricultural operations pay there, Jews are taking their place in great numbers, and are very successful. Mr. Arnold White also states that he has just returned from the Jewish agricultural colonies of Cherson and Ekaterinoslav, which number thirty thousand persons, and these live entirely by agriculture. Moreover, they are said to be industrious, sober, and remarkably free from vice, a statement which we can well believe. The truth is that the Jews are an intellectual, practical, virtuous, and industrious people; and instead of despising them, and striving to fight against them, modern nations would do well, not only to give them freedom and fair play, but also to learn the secret of their almost universal success.

British Fruit and British Farmers.

Dairy produce is not exactly fruit, though it is, in a sense, first cousin to it. The two were considered together by Mr. Chaplin at the Mansion House meeting on Monday. To those Englishmen who have practical experience of the questions raised at the meeting, Mr. Chaplin's figures were really grievous. Last year, said the speaker, we imported dairy produce to the value of £18,500,000. Before proceeding further let us consider this £18,500,000. This sum would have given in wages a pound a week to three hundred and fifty thousand men, that is to say, it would have supported three hundred and fifty thousand families each consisting of a husband, wife, and three or four children; in round numbers close upon two million persons. Mr. Chaplin contended that nearly all the dairy produce thus purchased from foreign sellers might have been produced at home. Last year we spent in foreign fruit £4,349,000. A third of the money thus spent we could have kept at home by producing our own fruits. So that in foreign fruit and dairy produce alone, we spend nearly £20,000,000 of money every year which might just as easily be spent in our own country. Somebody is at fault here; and it can only be one of three classes of persons. Either it is the landlords, or it is the farmers, or it is the labourers. Now we appeal to every fair-minded landlord and ask him: Does he in his heart consider it is the fault of the labourers? It certainly is not! No labourer can have a dairy without cows; and no man can keep cows without land. Where is the labourer's land? Where are even Mr. Chamberlain's three acres? Similarly, no labourer can produce fruit without an orchard; and no man can bring an orchard to fruit-bearing maturity without land, and permanent employment, and secure tenure. But where is the labourer's orchard land? And where are his permanent employment and his security of tenure? These things do not exist; they have yet to be created. Clearly, then, the blame cannot be put upon the labourer. But in that case it belongs either to the landlord or to the farmer. The actual truth perhaps is that both landlord and farmer are to be blamed, though the farmer by far the most. The British farmer, "taken in the lump," as somebody has put it, is not clever; and he is not enterprising. He will not work with his hands, and he cannot work with his brains. A man who works neither with brains nor with hands has usually but short shrift. Fruit growing, according to the English farmer's ideas, has something poor and paltry about it. Market gardeners are the proper persons to deal with it; the farmer is much too superior a creature for work of that kind. Similarly, dairying is not the sort of swaggering occupation which suits the taste of the breeder of shorthorns, or of the man who has been accustomed to sow extensive breadths of Autumn wheat. The farmer, poor ignorant blockhead, is a long way above all these unfashionable occupations. The landlord knows better. He is educated enough to understand that the most dignified farming is that which is most profitable. It would be well if some of the landlords would call their tenants together occasionally and instruct them in the elements of common sense and profitable agriculture.

